# The impact of nutrient deficiency on the structure of soil microbial communities within a double-cropping system

**DOI:** 10.3389/fpls.2025.1487687

**Published:** 2025-01-29

**Authors:** Rulan Yang, Zheng Sun, Yu Gong, Peng Zhou, Xinping Zhang, Jie Wang, Qiang Dong, Fei Gao

**Affiliations:** ^1^ College of Agronomy, Shanxi Agricultural University, Taiyuan, China; ^2^ Laboratory of Green Innovation, Advanced Institute of Natural Sciences, Beijing Normal University, Zhuhai, China

**Keywords:** bacteria, fungus, microbial community structure, summer maize, winter wheat

## Abstract

In the North China Plain, it is common for farmers to regularly clear crop residues from their fields. The prevalent practice of fertilization in this region continues to depend heavily on the use of compound fertilizers. Howere , long-term single fertilizer application has become the norm in the present agricultural production, which not only destroys the crop rotation system but also negatively affects the soil environment and crop yields. The current knowledge of how nutrient deficits affect the microbial community structure in double-cropping systems is still limited. To clarify the specific response of soil microorganisms to the absence of key nutrients in the ecosystems of the annual double cropping system, this study investigated how the lack of essential nutrients affected the diversity, abundance, and functional dynamics of microorganisms in the soil, and designed five treatment methods: (1) CK, nofertilizer treatment; (2) NPK, adequate nitrogen fertilizer, phosphorus fertilizer, and potassium fertilizer treatment; (3) PK, nitrogen deficiency treatment; (4) NK, phosphorus deficiency treatment; and (5) NP, potassium deficiency treatment. The results showed that in two growing seasons, NPK treatment increased the yields of wheat and corn by 16.9% and 27.0%, respectively, while NK and NP treatments increased by 13.4%, 5.4%, 25.0%, and 17.9%, respectively, and the total annual yield increased by 21.1%. In addition, NPK treatment promoted the microbial diversity and abundance of wheat and maize, and balanced fertilization provided more comprehensive nutritional support for crops. Compared to other nutrient-deficient treatments, NPK treatment substantially increased the abundance and functional diversity of soil bacterial and fungal communities (p<0.05). The structure and abundance of soil microbial communities are significantly correlated with soil physicochemical factors that involve organic matter, pH, potassium content, phosphorus, and nitrogen levels. pH is the primary environmental factor influencing the diversity of soil microbial communities.

## 1 Introduction

The system of winter wheat and summer maize double cropping is a crucial agricultural production mode in the North of China, which plays a significant role in China’s Food Security Strategy. However, a prolonged application of single and fixed fertilizers can lead to damage to the crop rotation systems, thereby impacting its production potential (Venkatesh et al., 2017). Currently, in the North China Plain, it is common for farmers to regularly clear crop residues from their fields. The prevalent practice of fertilization in this region continues to depend heavily on the use of compound fertilizers. Consequently, the depletion of essential soil nutrients over time hinders the soil’s capacity to support crop growth, potentially leading to reduce crop quality within rotational farming systems. Sustainable nutrient management practices are necessary to mitigate this risk (Zingore et al., 2006).

Soil microorganisms constitute an indispensable component of agroecosystems, influencing crop growth and soil quality through their participation in soil nutrient cycling (Geisseler and Scow, 2014). Within double-cropping systems, crops have distinct demands for soil nutrients, and rational fertilizer application strategies are beneficial for improving soil microbial activity (Laurent et al., 2023). Comprehending how these nutrients impact soil microbial communities is an essential prerequisite for achieving integrated soil–crop nutrient management. Soil functions as a “microbial reservoir,” offering a vast array of microbial options for plants (Mendes et al., 2015). Soil microorganisms participate in numerous soil material cycles and nutrient conversion processes, serving as a pivotal factor in the cultivation and maturation of crops (Bardgett and van der Putten, 2014; Jansson and Hofmockel, 2020). These microorganisms can swiftly and sensitively respond to alterations in the external environment. Soil microbial population richness, diversity, and community composition are important indicators for assessing soil fertility and agricultural ecosystem stability (Colomb et al., 2000; Geisseler et al., 2017). Microorganisms indirectly alter soil nutrient composition by influencing the rate and extent of organic matter decomposition and are important factors in the cycling of nutrients in the soil (Opoku et al., 2022; Wu et al., 2023). Moreover, soil nutrient composition, including the ratios of carbon, nitrogen, phosphorus, potassium, and other nutrition elements incorporated into the soil (Borase et al., 2020; Qiao et al., 2024), can impact microorganisms and thereby regulate crop productivity to some extent (Nannipieri et al., 2017; Wang et al., 2020).

Nutrient management strategies not only influence above-ground crop growth (Grove et al., 2004; Weber et al., 2007) but also significantly impact the biomass, community composition, prevalence, and activity of underground microbes in the soil (Delei et al., 2021). Different fertilizer combinations are important factors affecting soil physicochemical properties in crop rotation systems (Li et al., 2011). Meanwhile, the effects on soil microorganisms are more intricate. Other studies have demonstrated that the way microorganisms in crop rotation soils respond is linked to their functions within the microbial community of soil and the methods of fertilizer application (Venter et al., 2016; Breidenbach et al., 2017). Recent studies on the range of diversity and structural makeup of soil microbial communities in reaction to different fertilizer treatments are still being debated (Zhu et al., 2020; Shehryar et al., 2021). Extended field experiments have suggested that prolonged fertilizer use can alter soil properties (Bei et al., 2018) and potentially enhance soil microbial stability and increased microbial diversity in rice cultivation systems (Huang et al., 2019). Conversely, it has also been observed that fertilizer application in a single season can significantly alter the abundance of microbial communities in the soil (Zhang et al., 2022; Wei et al., 2023).

Crops manipulate the soil microbiome under nutrient-poor conditions; for example, maize plant roots regulate lateral root development and nitrogen capture by recruiting Oxalobacteraceae to enhance their performance under low-nitrogen environments (Peng et al., 2021). This reflects the role of soil microorganisms in coping with crop nutrient limitation. Moreover, different nutritional deficiencies significantly affect soil microbial communities, with bacterial species composition being more affected by nitrogen deficiency, while intraspecific interactions are mostly affected by phosphorus deficiency and least affected by potassium deficiency (Ma et al., 2013). Functional microorganisms involved in nitrogen fixation, nitrification, denitrification, and nitrate reduction carry out the majority of nitrogen cycling activities (Kuypers et al., 2018). Among them, nitrogen-fixing microorganisms, are an important functional group in plants and soil. These typically exist in rhizosphere ecosystems and play a crucial role in regulating plant nitrogen nutrition (Fierer et al., 2012). An imbalance of soil nutrients may result in the breakdown of soil aggregates and the disruption of microbial populations, with a detrimental impact on soil fertility and crop quality (Philippot et al., 2010). This is harmful to the sustainability of agricultural production. Overall, long-term single and fixed fertilizer application methods can cause damage to crop rotation systems, leading to insufficient soil fertility and subsequently affecting crop yields throughout the year. Therefore, gaining insight into the role that soil microorganisms play within the crop–soil system under varying conditions of nutrient deficiency is of great practical importance. Such understanding is crucial for refining and enhancing the fertilization strategies used in the North China Plain’s double-cropping systems, ultimately leading to more effective and sustainable agricultural practices in the geographical region.

The microbial community in the double-cropping systems has a substantial influence on the soil function of current and subsequent crops and farmland, playing an extremely important part in natural and agricultural systems (Li et al., 2021; Maria-Soledad et al., 2021). Compared with previous research that mainly focused on the nutrient treatment of single-season crops, our research focuses on the dynamics of microbial communities in the annual rotation system of winter wheat and summer maize in the field and explores the response of soil microorganisms to soil ecosystems under different nutrient deficiency conditions. Therefore, this study was conducted to analyze the effects of different nutrient deficiency conditions on the structure, diversity, and function of soil microbial communities in a double-season crop system. The specific objectives include 1) exploring the effects of different nutrient deficiency conditions on soil microorganisms under the winter wheat summer maize rotation system, 2) determining the impact of nitrogen, phosphorus, and potassium nutrient deficiencies on the construction of bacterial and fungal communities within the soil, and 3) clarifying the potential connection between soil microorganisms and physicochemical properties under nitrogen, phosphorus, and potassium nutrient deficiencies. The aim is to uncover the specific response of soil microorganisms to the lack of key nutrients in the annual double-cropping farmland ecosystem and to provide a theoretical basis and practical guidance for formulating effective nutrient management strategies suitable for the North China Plain region of China and promoting the development of sustainable agricultural.

## 2 Materials and methods

### 2.1 Study location and experimental design

The study location is situated in Baicun, a hamlet in Xinjiang County, which is part of Yuncheng City in Shanxi Province, central China. The location is positioned at coordinates of 35.67° N and 111.18° E, at a high point of 445 m above sea level. The region belongs to a warm temperate continental climate, which experiences an average yearly precipitation of 539.5 mm. **Supplementary Figure S1** shows the specific weather conditions during the growing season. The soil in this region has been identified a brown soil type.

**Figure 1:**
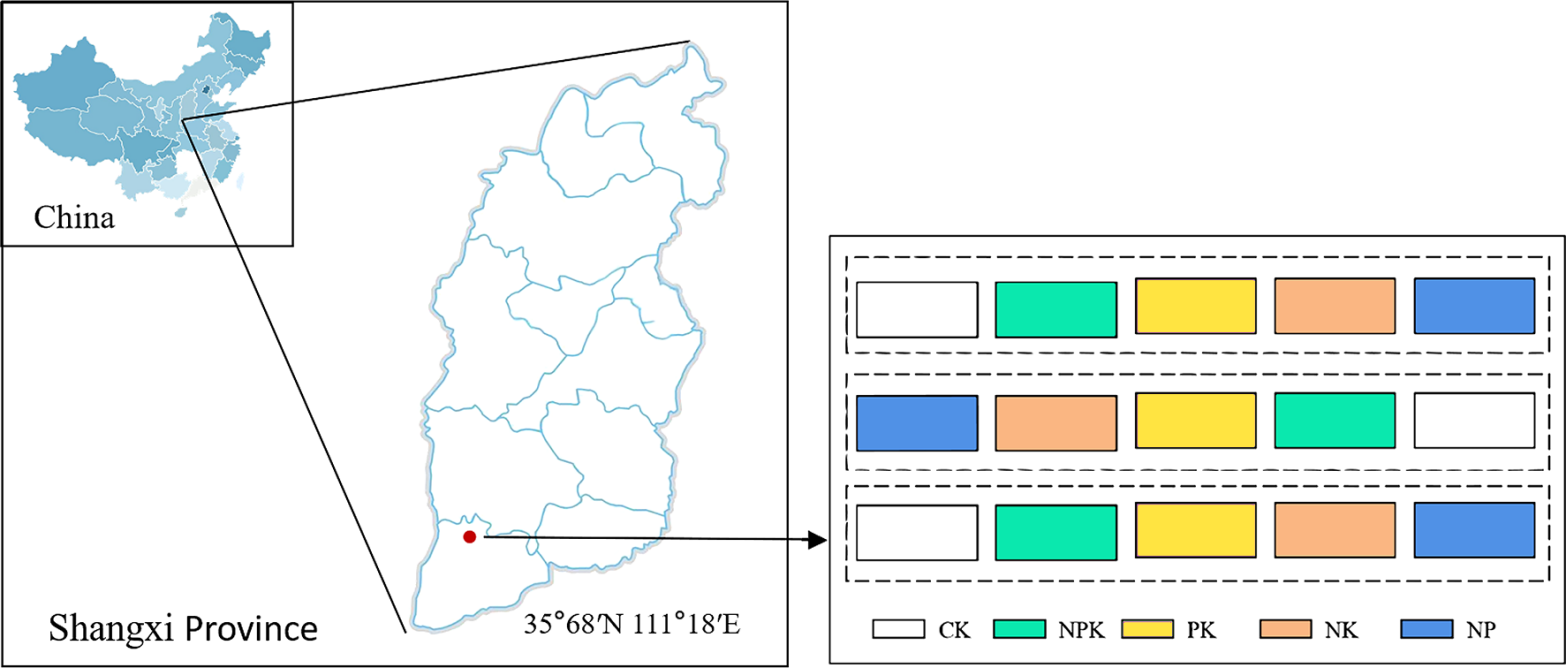
Sampling site and nutrient deficiency test setup.

This experiment adopted a double-season rotation mode of winter wheat summer corn, and five nutrient-deficient fertilization treatments were designed starting from the onset of the winter wheat season growing period that began in 2019. The trial used a randomized block design. Each treatment was set up for three repetitions. Yielding an aggregate of 15 plots, each with an area of 30 m^2^. Specific treatments include the following: (1) CK, no fertilizer treatment; (2) NPK, adequate nitrogen fertilizer, phosphorus fertilizer, and potassium fertilizer treatment; (3) PK, nitrogen deficiency treatment; (4) NK, phosphorus deficiency treatment; and (5) NP, potassium deficiency treatment. **Figure 1** depicts the experimental setup and layout of sampling points. The experimental plot was compacted by rotary tillage and then manually sown. The summer maize variety was Denghai 605, and the winter wheat variety was Malan No. 1. Urea with 46% nitrogen (N), superphosphate (P_2_O_5_ 15%), and potassium sulfate (K_2_O 50%) were used in this experiment, all of which were applied as a one-time basal fertilizer before sowing. Winter wheat for all plots in 2021–2023 was sown on 20 October and harvested on 15 June; summer maize for all plots in 2022–2023 was sown on 20 June and harvested on 15 October. Before planting winter wheat, the straw from the previous season’s summer corn was removed from the field. Similarly, to avoid and prevent nutrient accumulation caused by straw mixing and ensure soil nutrient balance, all of the wheat straw was taken out from the field before sowing summer corn. After being tilled and compacted by rotary tillers, summer corn and winter wheat are manually sown in the fields. All treatments were carried out using uniform agronomic measures, and specific nutrient allocation and cultivation management measures are shown in **Table 1**.

**Table 1:** Cultivation and management practices of different nutrient allocation.

Treatment		CK		NPK		PK		NK		NP	
		Wheat	Maize	Wheat	Maize	Wheat	Maize	Wheat	Maize	Wheat	Maize
Tillage method		Straw removal, manual rotary tillage									
Seeding rate (×104 seeds ha^−1^)		300	6.75	300	6.75	300	6.75	300	6.75	300	6.75
Seeding date (m/d)		10/20	6/20	10/20	6/20	10/20	6/20	10/20	6/20	10/20	6/20
Harvest date (m/d)		6/15	10/15	6/15	10/15	6/15	10/15	6/15	10/15	6/15	10/15
Fertilizer application rates (kg ha^−1^)	N	0	0	276	276	0	0	276	276	276	276
	P	0	0	67.5	67.5	67.5	67.5	0	0	67.5	67.5
	K	0	0	150	150	150	150	150	150	0	0

Before planting wheat and maize, soil samples were collected from each plot at a depth of 0–20 cm using a five-point sampling approach. In preparation for examination, the collected soil samples were air-dried, crushed, and sieved inside. These samples were then used to determine several soil characteristics such as pH, soil organic matter (SOM), total nitrogen (TN), total phosphorus (TP), total potassium (TK), available phosphorus (AP), and available potassium (AK). **Table 2** summarizes the experimental site’s basic soil parameters.

**Table 2:** Properties of the topsoil at the experimental site in 2021 and 2022.

Year	pH	SOM	TN	TP	TK	AP	AK	HN
		(g/kg)	(g/kg)	(g/kg)	(g/kg)	(mg/kg)	(mg/kg)	(mg/kg)
2021	8.57 ± 0.02	12.94 ± 0.11	0.96 ± 0.02	0.86 ± 0.05	15.74 ± 0.02	11.31 ± 1.98	113.6 ± 194.8	54.24 ± 44.87
2022	8.48 ± 0.27	13.05 ± 0.77	0.93 ± 0.01	0.75 ± 0.02	16.08 ± 0.26	10.9 ± 1.76	109 ± 71	63.5 ± 72.29

SOM, organic matter; TN, total nitrogen; TP, total phosphorus; TK, total potassium; AP, available phosphorus; AK, rapidly available potassium; HN, alkaline hydrolysis nitrogen.

### 2.2 The period and method of soil microbial sampling

A five-point method of sampling was utilized to gather surface soil samples (0–20 cm) from every site upon wheat and maize physiological maturity in 2022. To keep the newly acquired soil samples intact, they were immediately stored in an ice box. Impurities including stone debris and plant root residues were thoroughly removed from the samples. The soil was then sieved through a 1-mm screen to ensure homogeneity. The processed samples were sent back to the laboratory and kept at −80°C to preserve their quality for eventual DNA extraction.

### 2.3 The process of extracting DNA followed by sequencing with the Illumina MiSeq platform

DNA was isolated from the sample employing the CTAB/SDS technique, quality, and the concentration of DNA was assessed using a 1% agarose gel. The DNA was diluted with sterile water to a concentration of 1 ng/μl. The diluted genomic DNA served as a template for amplifying the V4 region of the bacterial 16S ribosomal RNA gene, using primers 515F (5′-CCTAYGGGRBGCASCAG-3′) and 806R (5′-GGACTACNNGGGTATCTAAT-3′). Primers ITS5-1737F(5′-GAAGTAAAAGTCGTAACAAGG-3′) and ITS2-2043R (5′-GCTGTGTTCATGATGC-3′) were used to target and amplify the ITS1 region in the sequencing of fungal ITS rRNA genes.

The PCR amplification method was as follows: initial denaturation at 98°C for 1 min, followed by denaturation at 98°C for 15 s, annealing at 50°C for 30 s, then extension at 72°C for 30 s, and finally extension at 72°C for 5 min. The PCR results were then examined by electrophoresis on a 2% agarose gel, allowing for the detection of the amplified fragments. The target DNA fragments were meticulously recovered and purified using the QIAquick Gel Extraction Kit (Qiagen, Germany). The purified amplicons were subjected to Illumina MiSeq sequencing by Beijing Novogene Technology Co., Ltd., Beijing, China with sequencing performed on the NovaSeq 6000 platform.

### 2.4 Bioinformatic analysis

Begin by performing preliminary processing on the raw data generated by the Illumina MiSeq sequencing platform. Initially, use FLASH software to concatenate the sequences of each sample. Following this, utilize the FsATP tool for rigorous filtration of the concatenated sequences, ensuring the removal of any chimeric sequences to yield effective tags suitable for further analysis. Subsequently, apply the Upaste algorithm (Upaste v7.0.1001) to cluster the validated tags across all samples, with sequences being grouped into operational taxonomic units (OTUs) under the standard 97% similarity criterion. For species annotation, use the Mothur method in conjunction with the SILVA138.1 SSU rRNA database to analyze the representative OTU sequences with the highest frequency, setting the threshold between 0.8 and 1. Finally, immediately multisequence match all OTU representative sequences using the MUSCLE (Version 3.8.31) program.

### 2.5 Statistical analysis

The data were processed using Microsoft Excel 2019 (Microsoft Corp., Redmond, WA, USA). SPSS 26.0 was used to run a one-way ANOVA. R software (version 2.15.3) was used to assess significant differences using Tukey’s *post-hoc* test (p<0.05) and Kruskal–Wallis rank sum test for data that did not match normal distribution. The alpha and beta diversity were calculated using QIIME (Version 1.9.1), and the Chao1 and Shannon indices were used to explore the alpha diversity of soil bacterial and fungal communities in the absence of particular components. Significance tests for differences in community structure among treatments were performed using ANOSIM. Principal coordinate analysis (PCoA) was conducted to evaluate differences in microbial community composition using weighted UniFrac distance, and the ggplot2 package in R software was used for visualization. Functional prediction of soil microorganisms was made under different nutrient deficiency treatments using FAPROTAX and FunGuild. Redundancy analysis (RDA) of different microorganisms and soil physicochemical properties was performed using the vif. cca function in the Vegan package of R software to determine the main physicochemical properties that affect community distribution. Heatmap Illustrator software was used to create heat maps, while other charts are created using R software (Version 2.15.3) and OriginPro (Version 2016, USA). OTU dilution curves of species diversity are shown in **Supplementary Figure S2**.

## 3 Results

### 3.1 Yield and composition of winter wheat and summer maize

Compared with the CK, the NPK treatment enhanced the yield of winter wheat by 21.5% and that of summer maize by 27.5%; the total annual production rose by 21.1%. Compared with the CK, the NK treatment raised the yields of winter wheat and summer maize by 13.5% and 25.3%, respectively, while the NP treatment increased the yields of winter wheat and summer maize by 5.5% and 17.9%, respectively. The PK treatment led to a considerable decline of the crop yield in both seasons (p<0.05), indicating that N and P were more efficient than K in boosting crop yield.

In terms of yield components, except for the NPK treatment, the NK treatment had the highest number of spikes and 1,000-grain weight among the CK and PK treatments. Compared with CK, the number of grains per spike in wheat season was increased by 22.7%, thousand kernel weight exhibited no notable discrepancy between the NK treatment and the CK, and the number of grains per spike and thousand kernel weight in maize season was increased by 6.6% and 8.6%, respectively (**Table 3**).

**Table 3:** Effect of nutrient deficiency on winter wheat–summer maize yield in 2022–2023.

Year	Treatment	Winter wheat				Summer maize				
		Harvest ear number (million ears ha^−1^)	Harvest ear	1,000-grain weight (g)	Yield weight (kg ha^−1^)	Harvest ear number (million ears ha^−1^)	Harvest ear	1,000-grain weight (g)	Yield weight (kg ha^−1^)	Annual yield (kg ha^−1^)
2022	CK	7.5ab	35.1d	45.0b	11,799c	4.3b	614.1b	287.4b	7,653c	19,452d
	NPK	7.7a	40.9a	42.6d	13,329b	5.1a	618.4ab	323.4a	10,202a	23,531a
	PK	7.6ab	39.5b	43.4c	12,936b	4.5ab	598.0b	267.5b	7,221c	20,157c
	NK	7.4b	41.0a	45.4b	1,3798a	4.8ab	649.4a	319.6a	9,925ab	23,723a
	NP	6.9c	37.7c	46.6a	12,079c	4.8ab	578.2c	316a	8,957b	21,036b
2023	CK	7.6a	31.7c	46.9a	11,347c	4.4b	599.8ab	320.6b	8,492b	19,839c
	NPK	7.5b	38.6b	47.4a	13,727a	4.8a	623.5ab	343.9a	10,309a	24,036a
	PK	6.9c	37.2c	40.0b	10,230d	4.5ab	589.7b	307.6b	8,065b	18,295d
	NK	6.5d	41.0a	46.7a	12,449b	4.6ab	644.7a	340.5a	10,256a	22,705b
	NP	6.8c	38.4b	47.2a	12,309b	4.6ab	633.1ab	345.1a	10,081a	22,390b

Values followed by a different small letter within a column are significantly different at 5% probability level.

### 3.2 The impact of various nutrient deficiency treatments on soil microbial alpha and beta diversity

**Figure 2:**
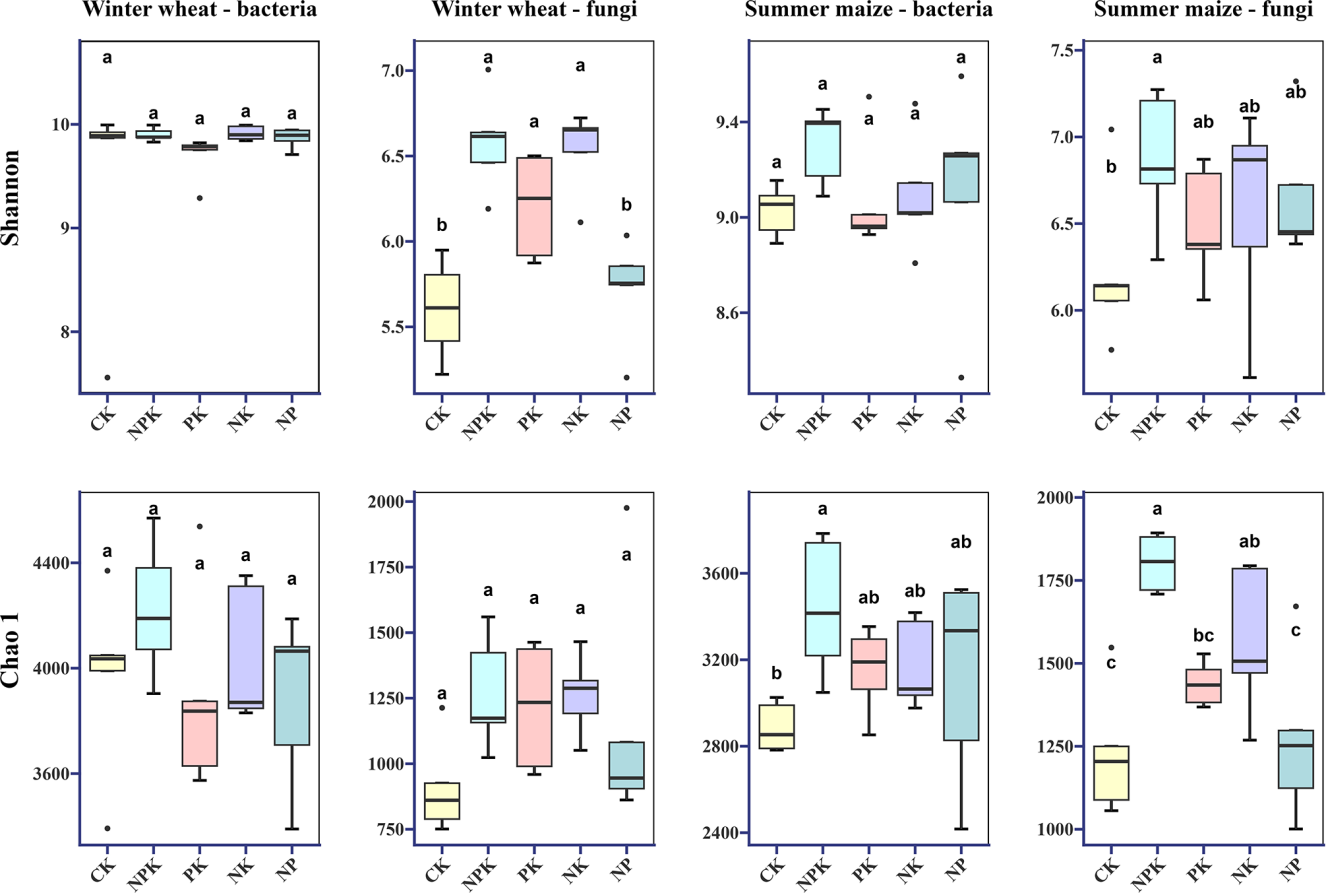
Alpha diversity of bacterial and fungal communities during growth in winter wheat and summer maize seasons under different nutrient deficiency treatments. Values followed by a different small letter within a column are significantly different at 5% probability level. Shannon and Chao1 index, alpha diversity.

In order to analyze the response of bacteria and fungi to different nutrient-deficient fertilization treatments, Shannon and Chao 1 indices, which can represent the diversity and richness of microbial communities, were selected (**Figure 2**), and the results showed that, in the winter wheat season, the Shannon and Chao 1 indices of bacterial communities were less responsive to nutrient deficiencies, and there was no significant difference between the treatments. The fungal community was more sensitive to potassium deficiency, and the Shannon index of NP treatment was significantly lower than that of NPK, PK, and NK treatments. In addition, the Chao 1 index under NP treatment was lower than that of NPK, PK, and NK treatments, but the difference was not significant. In the summer maize season, the Shannon index and Chao1 index of the bacterial community also did not show significant differences among the nutrient deficiency treatments. In contrast, the Chao1 index of the fungal community was significantly affected by PK and NP treatments, whereas the Shannon index did not show significant differences among the nutrient deficiency treatments.

In summary, the effects of different nutrient deficiency treatments on the α-diversity of bacterial and fungal communities showed similar trends in both seasons, with the highest α-diversity indices observed in both NPK treatments. Nutrient deficiencies resulted in lower colony diversity and richness compared to NPK treatments, especially under nitrogen- and potassium-deficient conditions.

The impact of various nutrient shortage treatments on microbial beta diversity was investigated using principal coordinate analysis (PCoA) based on a weighted unifrac matrix (**Supplementary Figure S3**) to analyze the distribution differences of bacterial and fungal communities. During winter wheat seasons, bacterial (**Supplementary Figure S3A**) and fungal (**Supplementary Figure S3B**) PCoA explained 85.79% and 49.54% of β diversity, respectively. During summer maize seasons, bacterial (**Supplementary Figure S3C**) and fungal (**Supplementary Figure S3D**) PCoA explained 66.26% and 55.62% of β diversity, respectively. The cumulative explanatory power of the four primary axes is >50%, indicating that they can represent the community composition characteristics of soil microorganisms at the phylum level. The results of ANOSIM analysis showed that NK and NP treatments significantly altered (p < 0.05) bacterial and fungal community distributions in both seasons compared to NPK treatment, where PK treatment did not significantly differentiate the distribution of bacterial communities in winter wheat season (**Supplementary Table S1**).

### 3.3 The impact of various nutrient deficiency treatments on soil microbial community composition

**Figure 3:**
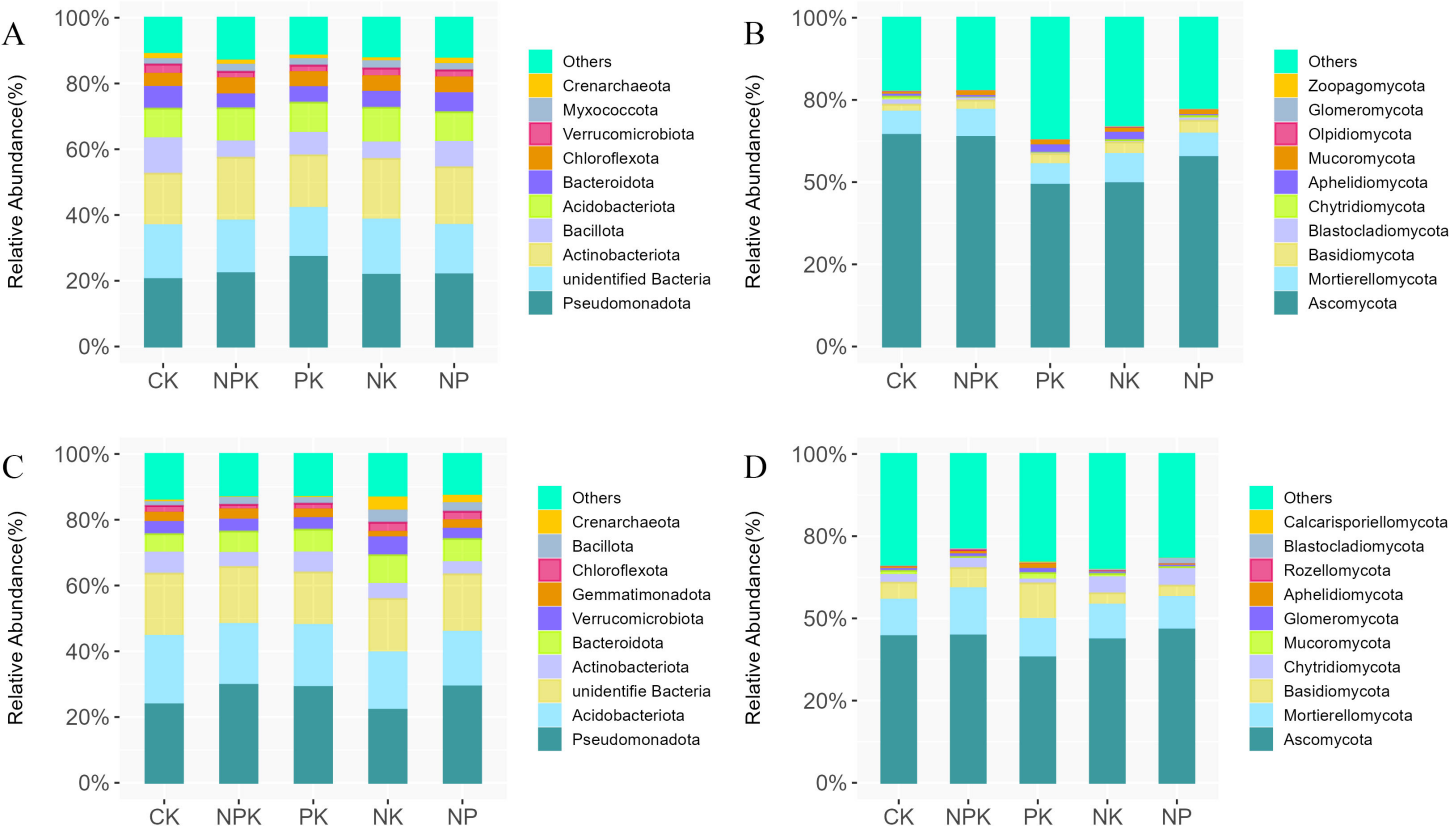
Effects of different nutrient deficiency treatments on the taxonomic composition of soil bacterial and fungal at the phylum level during the winter wheat and summer maize seasons Note: **(A)** represents the top 10 dominant bacterial species in the winter wheat season; **(B)** represents the top 10 dominant fungal species in the winter wheat season. **(C)** represents the top 10 dominant bacterial species in the summer maize season; **(D)** represents the top 10 dominant fungal species in the summer maize season. The relative abundance <1% replaced by Others.

We analyzed the changes in bacterial and fungal taxa at the phylum level and genus level and selected the top 10 phyla or genera based on average relative abundance (**Figure 3**; **Supplementary Figure S4**) First, at the phylum level (**Figure 3**), in the winter wheat season, Pseudomonadota was the most dominant phylum among all treatments and has the highest relative abundance (27%) under PK treatment. Other predominant phyla were unidentified Bacteria (17%), Actinobacteriota (16%), Acidobacteriota (10%), Bacillota (10%), and Bacteroidota (6%). In addition, PK treatment significantly increased the relative abundance of Pseudomonadota compared with NPK. NK and NP decreased the abundance of Pseudomonadota compared with PK (**Figure 3A**). The fungal community was dominated by Ascomycota (56%), Mortierellomycota (7%), and Basidiomycota (3%). The relative abundance of Ascomycota under NPK treatment was the highest (63%). Compared with NPK, the relative abundance of Ascomycota under PK, NK, and NP treatments decreased by 22%, 14%, and 6%, respectively. Compared with CK, the relative abundance of Aphelidiomycota increased significantly under PK and NK treatments, but not significantly under NPK and NP treatments (**Figure 3B**). In the summer maize season, the most dominant bacterial phyla were Pseudomonadota (26%), Acidobacteriota (18%), unidentified Bacteria (17%), and Bacteroidota (6%). Pseudomonadota were dominant and had the greatest relative abundance under NPK and NP treatments. We found that the relative abundance of Crenarchaeota increased significantly under NK and NP treatments compared with CK, with relative abundances of 3% and 2%, respectively, while the effects were not significant under NPK and PK treatments (**Figure 3C**). Similarly, in the summer maize season, the dominant phyla of the fungal community were Ascomycota (46%), Mortierellomycota (14%), and Basidiomycota (10%). Among them, the relative abundance of Ascomycota was the highest in NP treatment and decreased significantly under PK treatment. Compared with CK, NP treatment increased the relative abundance of Blastocladiomycota. Compared with NPK, the relative abundance of Ascomycota under PK treatment was significantly reduced, and the relative abundance of Aphelidiomycota was significantly increased (**Figure 3D**).

Second, under nutrient deficiency conditions, species annotation was done on the OTUs obtained from various soil samples at different taxonomic levels, and the top 10 genera with clear annotation information and relative abundance were selected as dominant genera (**Supplementary Figure S4**). In the bacterial community of winter wheat season, the dominant bacterial genera were *Sphingomonas* and *Pseudomonas*. Compared with CK the relative abundance of *Pseudomonas* increased under NPK, PK, and NK treatments, and the relative abundance of *Pseudomonas* in PK treatment was the highest, with a relative abundance of 7%. Compared with NPK, the relative abundance of *Sphingomonas* decreased under PK treatment, while that of *Streptococcus* increased. The relative abundance of *Bacillus* was maximum (20%) under NP treatment (**Supplementary Figure S4A**). The relative abundance of *Fusicolla* increased, and the relative abundance of *Lophotrichus* and *Chaetomium* decreased significantly in the NPK treatment compared to CK. The relative abundance of *Chaetomium* increased significantly under PK treatment compared to NPK. The relative abundance of *Fusarium*, *Alternaria*, and *Mortierella* increased under NPK, PK, NK, and NP treatments compared to CK, with *Fusarium* and *Alternaria* having the highest relative abundance under the NP treatment and *Mortierella* having the highest relative abundance under the NK treatment. Compared with CK, the relative abundance of unidentified *Mortierellales* sp. in all treatments increased, and there were more unidentified *Mortierellales* sp. in NK treatment (**Supplementary Figure S4B**). In the summer corn season, we can see that the most dominant bacterial genus was *Sphingomonas*. Compared with CK, the relative abundance of *Sphingomonas* increased significantly under NP treatment. In addition, we found that the *Pseudomonas* increased significantly under NP treatment compared with NPK. The relative abundance of *Candidatus Nitrocosmicus* also increased significantly under NK treatment compared with CK (**Supplementary Figure S4C**). For fungal community, the relative abundance of *Aspergillus* and unidentified *Ascomycota* sp. increased significantly in NPK treatment but decreased in NK and NP treatments compared with CK. The relative abundance of *Rhizophlyctis* and *Chaetomium* increased significantly in NK and NP treatments and decreased in NPK and PK treatments compared with CK. The relative abundance of *Cladosporium* decreased under NK and NP, but increased in NPK and PK compared with CK (**Supplementary Figure S4D**).

### 3.4 The impact of nutrient deficiency on the functional dynamics of soil microbial communities

#### 3.4.1 Predictive analysis of bacterial community functions

**Figure 4:**
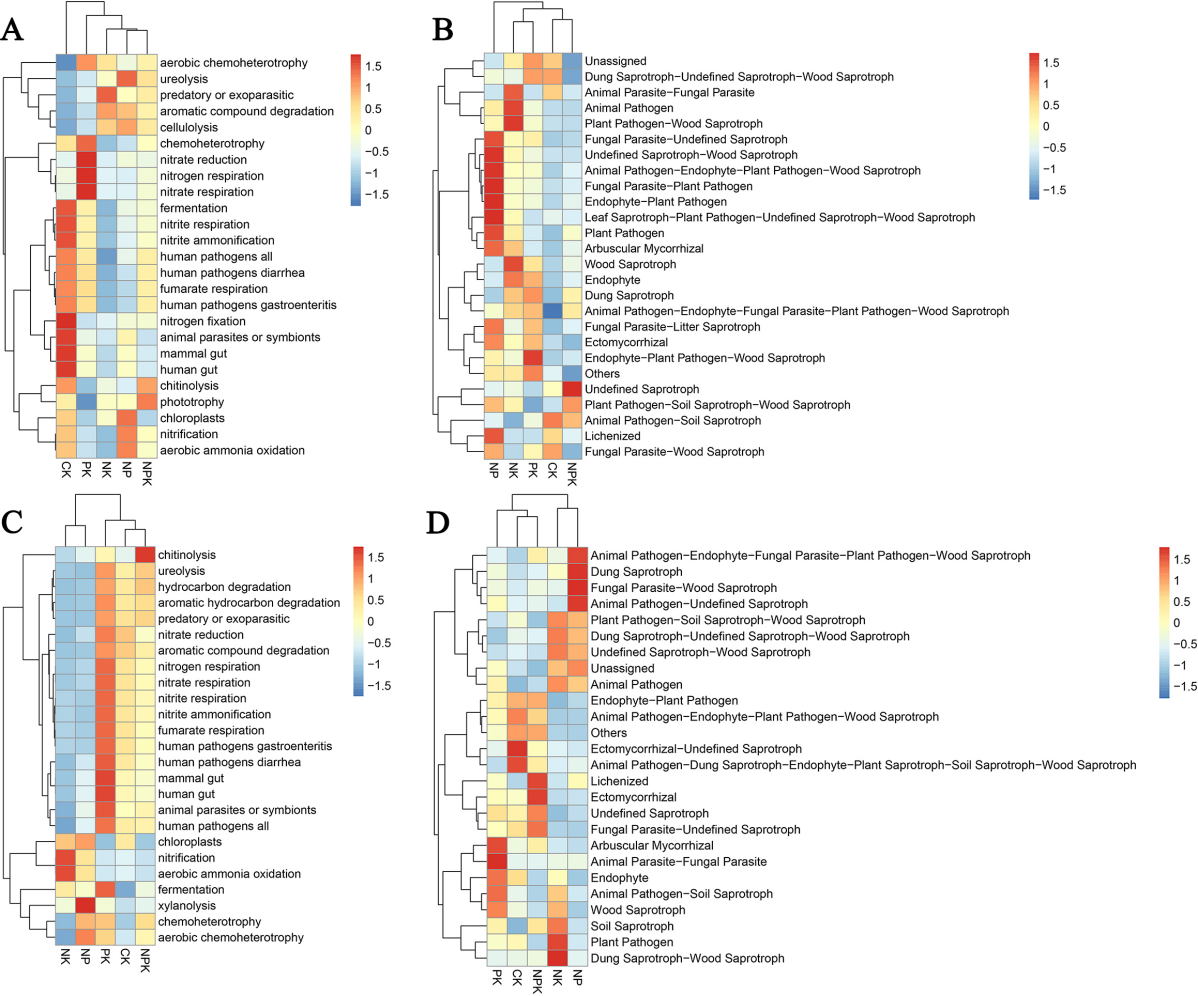
Microbial functional groups predicted by FAPROTAX and FUNGuild. Note: **(A)** represents the bacterial soil microbial functions during the winter wheat season; **(B)** represents the fungus soil microbial functions during the winter wheat season; **(C)** represents the bacterial soil microbial functions during the summer maize season; **(D)** represents the fungus soil microbial functions during the summer maize season.

Based on FAPROTAX, the proportion of prediction functions of the top 25 most abundant genes in different treatments was calculated (**Figures 4**). During the winter wheat season (**Figures 4A**), PK increased Chemoheterotrophy, nitrate reduction, nitrogen respiration, and nitrate-respiration-associated functional bacteria compared to CK. The NK treatment predominantly featured predatory or ectoparasitic, while the NP treatment notably enhanced functions such as ureolysis, chloroplasts, nitrification, and aerobic ammonia oxidation, where nitrification and aerobic ammonia oxidation mean release of nitrogen available for plant uptake. The functional bacteria related to nitrogen fixation was significantly reduced in each nutrient deficiency treatment. Nutrient deficiency reduced the soil’s demand for nitrogen fixation function, thus inhibiting the activity of nitrogen fixing microorganisms. Compared with PK, NK, and NP, chitinolysis increased under NPK treatment. In addition, we found that cellulolysis was significantly enhanced in NK, NPK, and NP treatments, whereas the effect of cellulolysis under PK treatment was not significant due to the fact that nitrogen deficiency may have inhibited the activity of certain cellulose-degrading-based flora. The results of PCA analysis showed that the functions of microbial communities were similar across treatments (**Supplementary Figure S5A**).

During the summer maize season (**Figure 4C**), the soil microbial community’s nitrification and aerobic ammonia oxidation effects were shown to be stronger in the NK treatment. Xylanolysis, chemoheterotrophy, and aerobic chemoheterotrophy functional bacteria were enhanced by the NP treatment, and chitinolysis was significantly enhanced by the NPK treatment, compared to CK. Obviously, the functional bacteria associated with pathogens (e.g., human pathogens gastroenteritis and human pathogens diarrhea), complex organic matter degrading functional bacteria (e.g., hydrocarbon degradation and aromatic hydrocarbon degradation), and N cycling related (nitrate reduction, nitrogen reduction, and nitrate respiration) by some functional bacteria, which were particularly significant in the PK treatment. In PCA analysis, the community distances among CK, PK, and NPK treatments were closer, indicating more similar compositions of microbial community functions (**Supplementary Figure S5C**).

#### 3.4.2 Functional annotation of fungus communities

The FUNGuild database was used to classify the nutritional kinds of soil fungus communities treated with different nutrient elements. It was discovered that the nutritional types of fungus communities in wheat and maize after harvest were saprotroph, pathotroph, pathotroph–symbiotroph, pathotroph–saprotroph, and pathotroph–saprotroph–symbiotroph. The fungus with facultative functions, such as saprotroph, was predominant (**Figure 4**).

More detailed microbial community ecological functions for each treatment were subsequently obtained from the FUNGuild database. During the winter wheat season (**Figure 4B**), the soils showed an increase in animal parasite–fungal parasite (APF)-related microbial community activity in the NK treatment compared with the other nutrient-deficient treatments, showing a positive response, and the PK treatment also showed a significant increase in endophyte–plant pathogen–wood saprotroph activity. However, the NP treatment had a significant positive effect on most microbial communities. Under nutrient deficiency treatments of NP and PK, the functions of ectomycorrhizal was significant, which plays an important role in promoting plant growth and development, enhancing host resistance, and improving the structure of the soil microbial community. Ectomycorrhizal also contributes by decomposing organic matter and promoting nutrient cycling. Additionally, functional groups such as dung saprotroph of PK treatment and wood saprotroph of NK treatment were more significant, and they had similar functions, including the decomposing of organic matter and promoting nutrient cycling.

After the maize season harvest (**Figure 4D**), the PK treatment prominently featured ecological functional groups such as arbuscular mycorrhizal fungi and animal parasite–fungi parasite. In the NP treatment, groups like dung saprotroph and fungal parasite–wood saprotroph showed higher responses, indicating that the absence of potassium, combined with nitrogen and phosphorus, positively affects the growth and metabolic activities of these fungal communities. The activities of fungi such as plant pathogen and dung saprotroph–wood saprotroph were enhanced under the NK treatment, likely due to the combined effects of nitrogen and potassium on their decomposition and nutrient absorption in woody environments. Similar to the full nutrient treatments in the wheat season soil, most fungal communities under the NPK treatment exhibited significant activity increases, especially the ectomycorrhizal. Fungal community function in the CK and NPK treatments in both seasons was more similar in composition than the other nutrient-deficient treatments (**Supplementary Figures S5B, D**)

### 3.5 Effects of environmental factors on soil microbial communities under nutrient deficiency conditions

#### 3.5.1 Environmental factors affecting the distribution of soil microbial communities

**Figure 5:**
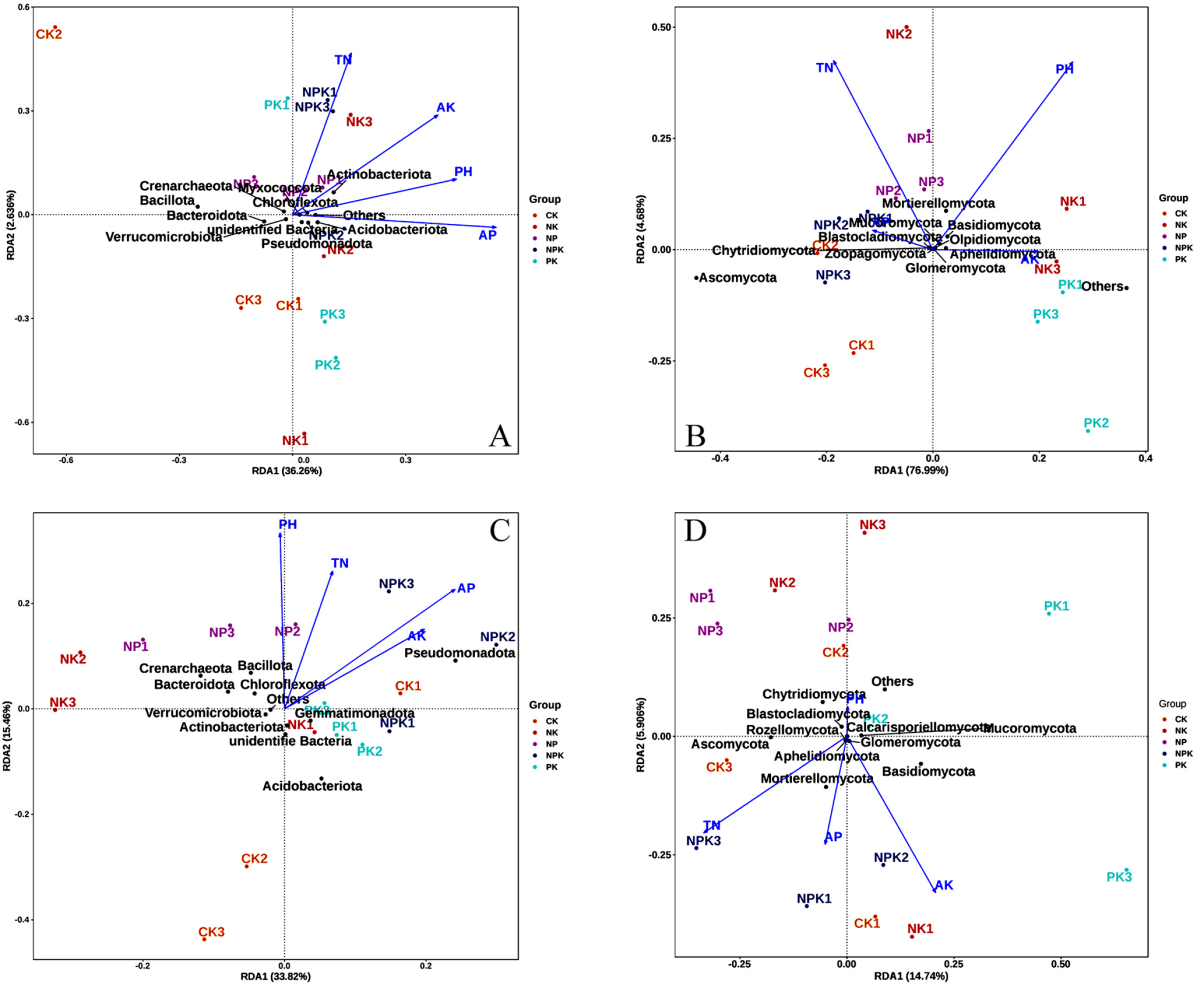
RDA analysis of soil bacterial and fungus communities and environmental factors in winter wheat and summer maize seasons at the phylum level. Note: **(A)** represents the bacterial phyla in the winter wheat season; **(B)** represents the fungal phyla in the winter wheat season. **(C)** represents the bacterial phyla in the summer maize season; **(D)** represents the fungal phyla in the summer maize season. TN, total nitrogen; AP, available phosphorus; AK, hydrolysis nitrogen.

The impact of soil physicochemical parameters on the distribution of bacterial and fungal communities in two seasons of soil under nutrient-deficit circumstances was investigated using high-throughput redundancy analysis (RDA). The findings revealed that four important soil physicochemical parameters influenced the spread of bacterial and fungal communities throughout two seasons. The association between soil physicochemical factors and phylum level microbial populations during the winter wheat–summer maize growing season is shown in **Figure 5**.

From the results, it was learned that pH, TN, AP, and AK are the key parameters affecting the distribution of soil bacterial and fungal communities (**Figure 5**). First, during the winter wheat, in the RDA analysis of bacterial communities, there is a positive correlation between various environmental factors such as pH, total nitrogen, available phosphorus, and available potassium, as the arrows between them form acute angles (<90°). Among them, the environmental variables AP and PH are the main factors affecting the distribution of bacterial communities in wheat season (**Figure 5A**). The effects of TN and PH on fungal community structure were more obvious. pH was negatively correlated with total nitrogen, as the arrows between them formed obtuse angles (**Figure 5B**).

During the summer maize, in the RDA analysis of bacterial communities, there was also a positive correlation between all environmental factors. Among them, PH was positively correlated with Bacillota, Crenarchaeota, Bacteroidota, and Chloroflexota (**Figure 5C**), while in the RDA analysis of fungal communities (**Figure 5D**), pH and other environmental factors showed a negative correlation. In summary, the results of RDA indicate that pH is a major physicochemical factor significantly influencing the distribution of bacterial and fungal communities, with the greatest explanatory power (p<0.05).

#### 3.5.2 Correlation analysis of microorganisms and physicochemical properties

**Figure 6:**
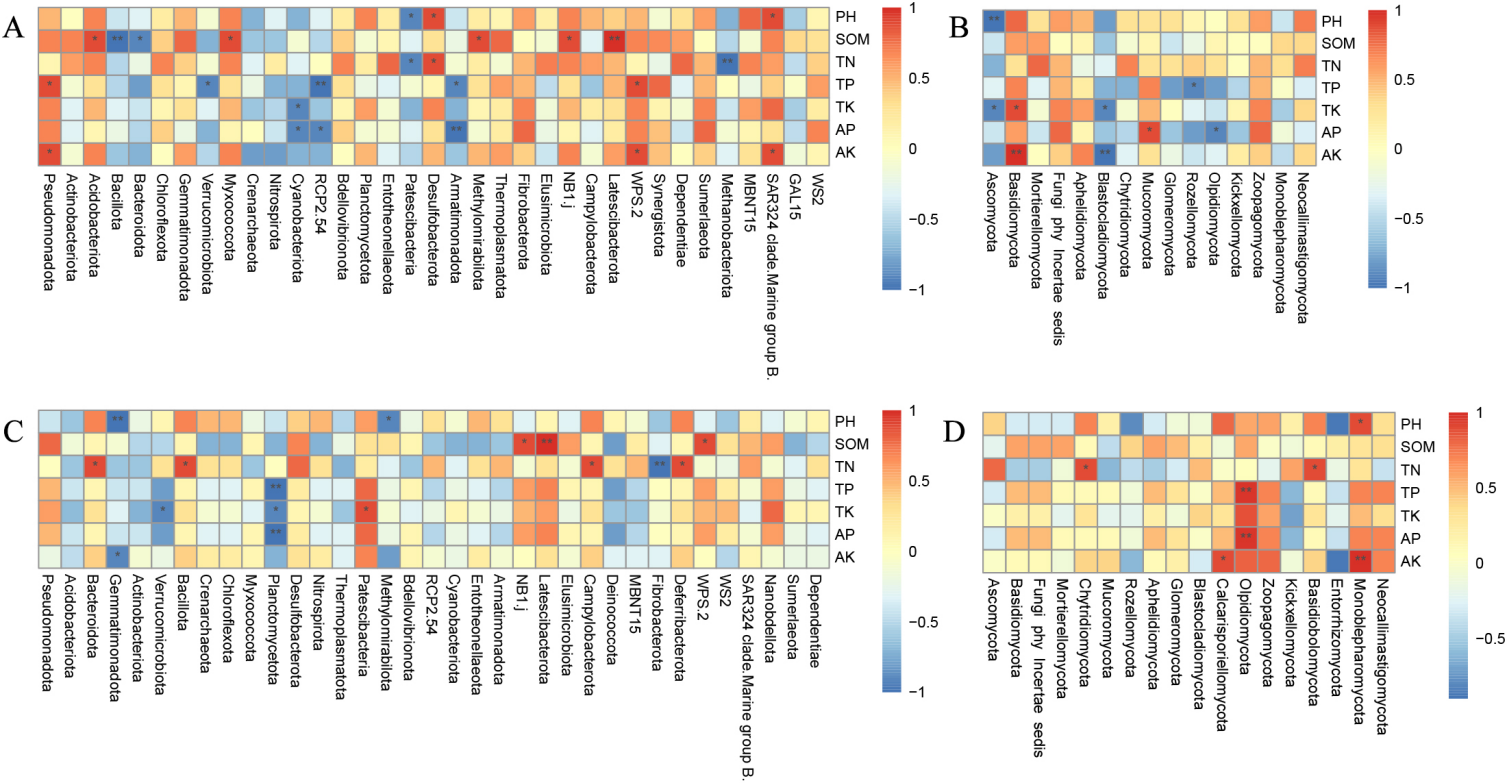
Heat map of correlation between soil bacterial and fungus communities and environmental factors in winter wheat and summer maize seasons. Note: **(A)** represents the bacterial phyla in the winter wheat season; **(B)** represents the fungal phyla in the winter wheat season. **(C)** represents the bacterial species in the summer maize season; **(D)** represents the fungal phyla in the summer maize season. X- and Y-axes are for different phyla and environmental factors, respectively. Spearman’s correlation coefficient R-value and significance p-value were calculated; R-value is shown in different colors in the graphs, while P-value is shown with an asterisk. *p<0.05, **p<0.01.

Through Spearman’s correlation analysis, the relationship between the dominant bacterial genera of soil microorganisms and environmental factors was further analyzed. The findings indicate that pH significantly affects the majority of bacterial communities in both seasons, typically showing a positive correlation. In contrast, the impact on fungal communities was less significant, with negligible correlations (**Figure 6**).

During the winter wheat season, correlation analyses between soil physicochemical characteristics and bacterial taxa revealed that SOM correlates positively with most bacterial groups, particularly showing highly significant positive relationships with Latescibacterota, and significant positive relationship with NB1j, Methylomirabilota, Myxococcota, and Acidobacteriota (p < 0.05). Conversely, there were significant negative correlations with Bacillota and Bacteroidota (p < 0.05). pH was positively correlated with SAR324 clade. Marine group B., it is particularly significant. Both TP and AK showed a positive correlation with Pseudomonadota, whereas TP was negatively associated with Verrucomicobiota (**Figure 6A**). Regarding fungal communities, SOM was similarly positively correlated, although the impact of TP and TN was more dispersed. In the fungal communities, Ascomycota showed a substantial negative association with TK (p < 0.05). Blastocladiomycota had a significant negative correlation with AK (p < 0.01) (**Figure 6B**).

During the summer maize season, almost all bacterial communities positively correlated with SOM, TN, and TP. The correlations with TK and AP, however, differed among groups, showing both positive and negative trends. Notably, Latescibacterota had an exceptionally strong positive correlation with SOM (p < 0.01). Other bacteria, including Gemmatimonadota, Bacillota, Campylobacterota, and Deferribacterota, had significant positive relationships with TN (p < 0.05). Planctomycetota had substantial negative associations with AP (p < 0.05), TK, and AK (p < 0.01) (**Figure 6C**). Fungi Olpidiomycota showed substantial positive correlations with TP and AP (p < 0.01), whereas Calcarisporiellomycota and Monoblepharomycota exhibited significant positive correlation with AK (p < 0.05) (**Figure 6D**).

## 4 Discussion

Crop yield and nutrient elements in soil are closely intertwined. In this study, nutrient-balanced NPK treatments significantly increased crop yields, and nutrient deficiencies led to a significant reduction in crop yield components carried out to affect total crop yields. Previous studies have shown that moderate application of chemical nitrogen fertilizers can improve soil nutrient supply and thus increase crop yield. In this study, there was no significant difference between NK and NP treatments in terms of total yield; that is, the difference in total crop yield between phosphorus- and potassium-deficient environments was not significant, but potash fertilizers had a more pronounced effect on the yield components than phosphorus fertilizers (Hou et al., 2015). In addition, the application of potash fertilizer can increase the number of spikes, spike length, and thousand grain weight (Rufty et al., 1988). In this study, when the application of N and K fertilizers reached the appropriate amount and the simultaneous increase in phosphorus fertilizer application significantly increased the crop yield components and grain quality, this increase in yield was mainly attributed to the comprehensive N, P, and K that provided the soil with an adequate supply of soil nutrients, which significantly increased crop productivity, a result that coincides with those of other authors (Wang et al., 2015; Weng et al., 2023). On the other hand, the different limitations on crop growth and development due to NPK deficiency may also be responsible for the yield differences (Ma et al., 2020).

Microorganisms are one of the most important indicators of soil health, and their presence and diversity can reflect the overall condition of the soil, including nutrient content, organic matter, and ability to support plant growth (Liang et al., 2015). Microbial diversity is closely related to nutrient cycling and soil function (Zheng et al., 2019; Jiao et al., 2021). Its community structure responds differently to different fertilization measures (Bruggen et al., 2015), and related studies have shown that nitrogen fertilizer application has different effects on soil bacterial diversity and their communities in different ecosystems (Coolon et al., 2018; Zhong et al., 2015). The diversity of bacterial and fungal communities also varies depending on the type of nutrient deficiency (Meena et al., 2017). In this study, nutrient-balanced NPK treatments generally had a positive effect on microbial diversity and abundance in winter wheat and summer maize, whereas nutrient deficiencies affected bacterial community diversity and abundance, especially under nitrogen- and potassium-deficient conditions. In addition, we found that bacteria had higher abundance and evenness than fungi in both seasons, and this difference may be due to the fact that bacteria are the dominant microorganisms in the soil, with faster responses and shorter turnover times to environmental changes (Usher, 2006), or it may be due to the fact that nutrient limitation reduces the availability of energy sources and substrates for growth, which leads to a reduction in the diversity of fungal microbial species (Griffiths et al., 2000). The C/N of bacteria and fungi varies depending on the nutrient requirements of the two, with C/N ratios of bacterial biomass ranging from 3 to 12, whereas fungal C/N ratios are expected to range from 3 to 60 (Strickland and Rousk, 2010), which, on average, implies that the C/N ratio of bacteria is lower than that of fungi, and therefore, bacteria may be more likely to have a higher nitrogen requirement.

Soil microbial community is an important and sensitive indicator of soil quality and a key biological indicator for evaluating the sustainability of soil ecosystems. Studies have shown that different fertilization treatments significantly affect the distribution of microbial communities by altering soil chemistry, e.g., long-term application of chemical fertilizers has a significant effect on the composition of soil fungal communities (Chinnadurai et al., 2014; Li et al., 2018; Wang et al., 2024). In addition, different species of bacteria and fungi in the microbial community have moderating responses to nutrient element deficiencies (Eva et al., 2021). These effects are not only related to the microbial community composition but also further affect the nutrient cycling and ecological functions of the soil. In this study, nutrient availability deficiencies significantly affected the composition of soil bacterial and fungal communities. At the phylum level, Pseudomonadota was the most dominant bacterial phylum in the soil, but its abundance was significantly reduced under NK and NP treatments. On the other hand, the fungal community was mainly composed of three taxa: Ascomycota, Mortierellomycota, and Basidiomycota. Among them, Ascomycota was the dominant taxon in the soil samples, a finding that is in agreement with previous studies (Ma et al., 2012; Liu et al., 2021). In genus level analysis, *Sphingomonas* was a dominant bacterium with good organic matter degradation ability. However, its relative abundance was significantly lower under PK treatment than NPK, NK, and NP treatments, suggesting that nitrogen deficiency may inhibit the growth of *Sphingomonas* and thus reduce its relative abundance in the community. In addition, soil bacterial and fungal community structure also showed significant differences between planting seasons in this study, which may be related to seasonal differences in plant root secretions, changes in nutrient requirements, and environmental conditions (Ashworth et al., 2017; Favela et al., 2024). In this study, nutrient deficiencies significantly affected the proliferation patterns of microorganisms in soil, which in turn altered their abundance and community structure. Bacterial and fungal populations are more diverse and stable in nutrient-rich soils, while nutrient-deficient conditions may lead to the loss of some key microbial taxa, thus affecting soil ecosystem functioning.

The functions of soil microorganisms are the central basis for maintaining soil homeostasis and facilitating the transformation and movement of multiple nutrients. The results of this study indicate that nutrient-sufficient NPK treatments have more significant promotion effects on the ecological functions of soil bacteria, especially in terms of nitrogen cycling, organic matter decomposition, and potential impacts on human and animal health. Specifically, nitrogen-cycle-related functions (e.g., nitrification and ammonia oxidation) and organic matter decomposition functions (e.g., chitinolysis and aromatic compound degradation) were significantly enhanced under nutrient-sufficient conditions. Meanwhile, under nutrient-sufficient conditions, soil bacteria significantly promote organic matter decomposition and nutrient release by decomposing organic matter for energy and nitrogen sources (Williams et al., 2013; Qin et al., 2015). Such processes further support the activity of oxidative-demanding heterotrophic microorganisms, which enhances the function of nutrient cycling in the soil, including nitrogen fixation, phosphorus release, and degradation of complex organic compounds (Chen et al., 2020). In addition, functional microorganisms in soil showed higher stability and diversity under NPK treatment, indicating that microbial communities under nutrient-sufficient conditions were able to respond and adapt to environmental changes more efficiently. In contrast, nutrient-deficient conditions limited the growth and metabolism of functional microorganisms and weakened the nutrient conversion efficiency of the soil. For example, the abundance of some key microorganisms was significantly reduced under nitrogen- or phosphorus-deficient conditions, leading to weakened nitrogen cycling and phosphorus solubilization functions (Zhong et al., 2020; Dang et al., 2022).

The distribution of microbial communities is closely related to soil physicochemical properties, and related studies have shown that the number of soil microorganisms is positively correlated with soil organic matter and nutrient contents (e.g., total nitrogen, quick-acting nitrogen, total phosphorus, and quick-acting phosphorus), but the number of actinomycetes and fungi is weakly correlated with these nutrients (Shi et al., 2020). In addition, microbial communities were more abundant and diverse in nutrient-balanced soils (Hou et al., 2017), suggesting that nutrient sufficiency is closely related to the number and diversity of soil microbial communities. In this study, it was observed that the community structure and abundance of soil microorganisms showed significant associations with physicochemical attributes such as soil organic matter, nitrogen, phosphorus, and potassium content, and pH, with soil pH being an important driver of changes in microbial communities, a finding that coincided with other studies, suggesting that nutrient availability and nutrient limitation have a high degree of dependence on soil microbial impacts (Fierer and Jackson, 2006; Ho et al., 2014). Available nitrogen and available potassium in the soil are considered to be key factors in explaining the total number of bacteria, actinomycetes, and fungi, whereas nitrogen deficiency may directly inhibit bacterial growth (Chau et al., 2005), and the populations of actinomycetes and fungi are more susceptible to limitation by the level of available potassium in the soil. This suggests that different types of microorganisms differ in their sensitivity to nutrient requirements and that their proliferation is significantly affected by nutrient supply patterns (Li et al., 2014; Young et al., 2018). In addition, nutrient limitation may also have profound effects on soil ecological functions by indirectly affecting the metabolic activities and biodiversity of soil microorganisms, e.g., N and K limitation not only reduces the abundance of specific functional microorganisms but also may alter microbial interactions and their roles in nutrient cycling (Thiele-Bruhn et al., 2012). Therefore, rational nutrient management strategies are essential for maintaining the abundance and diversity of soil microorganisms and the health of soil ecosystems.

## 5 Conclusions

Among different treatments, comprehensive analysis showed that NPK treatments significantly increased total yield output in winter wheat and summer maize by 21.1%. Nutrient deficiencies can lead to unmet nutrient needs during the nutritive growth stage of the crop, thus affecting the formation of yields at maturity. In addition, NPK treatment was most effective in maintaining the relative abundance of bacterial and fungal populations in general, with bacterial diversity being more affected in the winter wheat season and fungal diversity being more pronounced in the summer maize season. Nutrient-balanced treatments raised the relative abundance of Ascomycota, while nitrogen and phosphorus deficiency treatments lowered it. Meanwhile, the primary metabolic functions of microorganisms were also regulated by different nutrient deficiencies, especially the nitrogen cycling mechanisms, such as ammonia oxidation and nitrification, which were significantly promoted. The physicochemical property distribution of bacterial and fungal populations was considerably influenced by soil pH, particularly under nutrient deficiencies with the highest explanatory rate. These findings not only expand our understanding of how soil community structure and microecological functions respond under nutrient deficiency conditions but also provide a theoretical basis for optimizing fertilization schemes and maintaining the stability of farmland ecosystems in the North China Plain.

## Data Availability

The datasets presented in this study can be found in online repositories. The names of the repository/repositories and accession number(s) can be found below: https://www.ncbi.nlm.nih.gov/, PRJNA1212476.
