# Supplementary material for: The impact of nutrient deficiency on the structure of soil microbial communities within a double-cropping system
Source: Front Plant Sci. 2025 Jan 29;16:1487687. doi: 10.3389/fpls.2025.1487687 (PMC11814463; doi:10.3389/fpls.2025.1487687)
Supplement: Supplementary file 1 [file DataSheet1.docx]

**Supplementary materials**

**1 Supplementary Figures and Table**

- 1. **Supplementary Figures**

**Graphical abstract：**

**
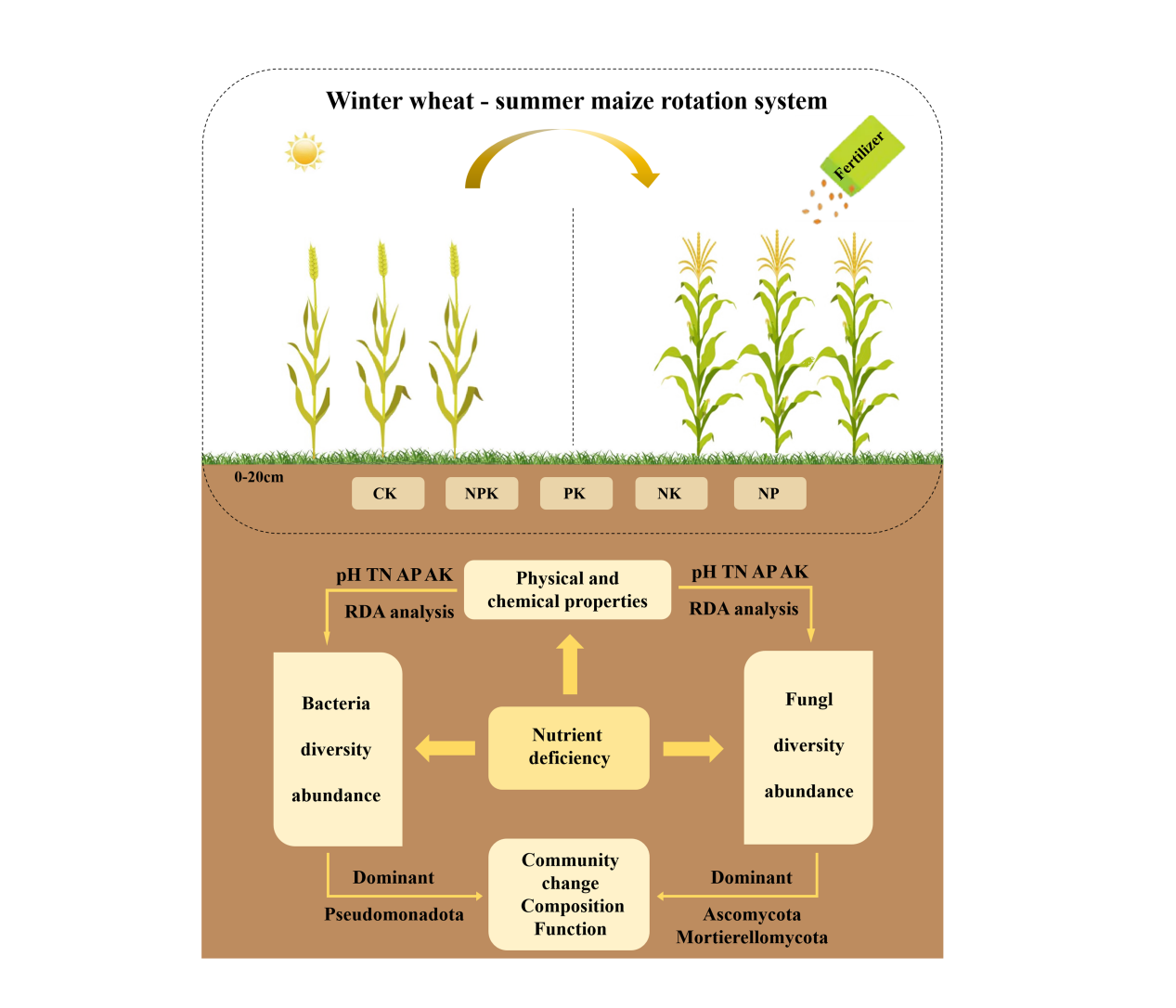
**


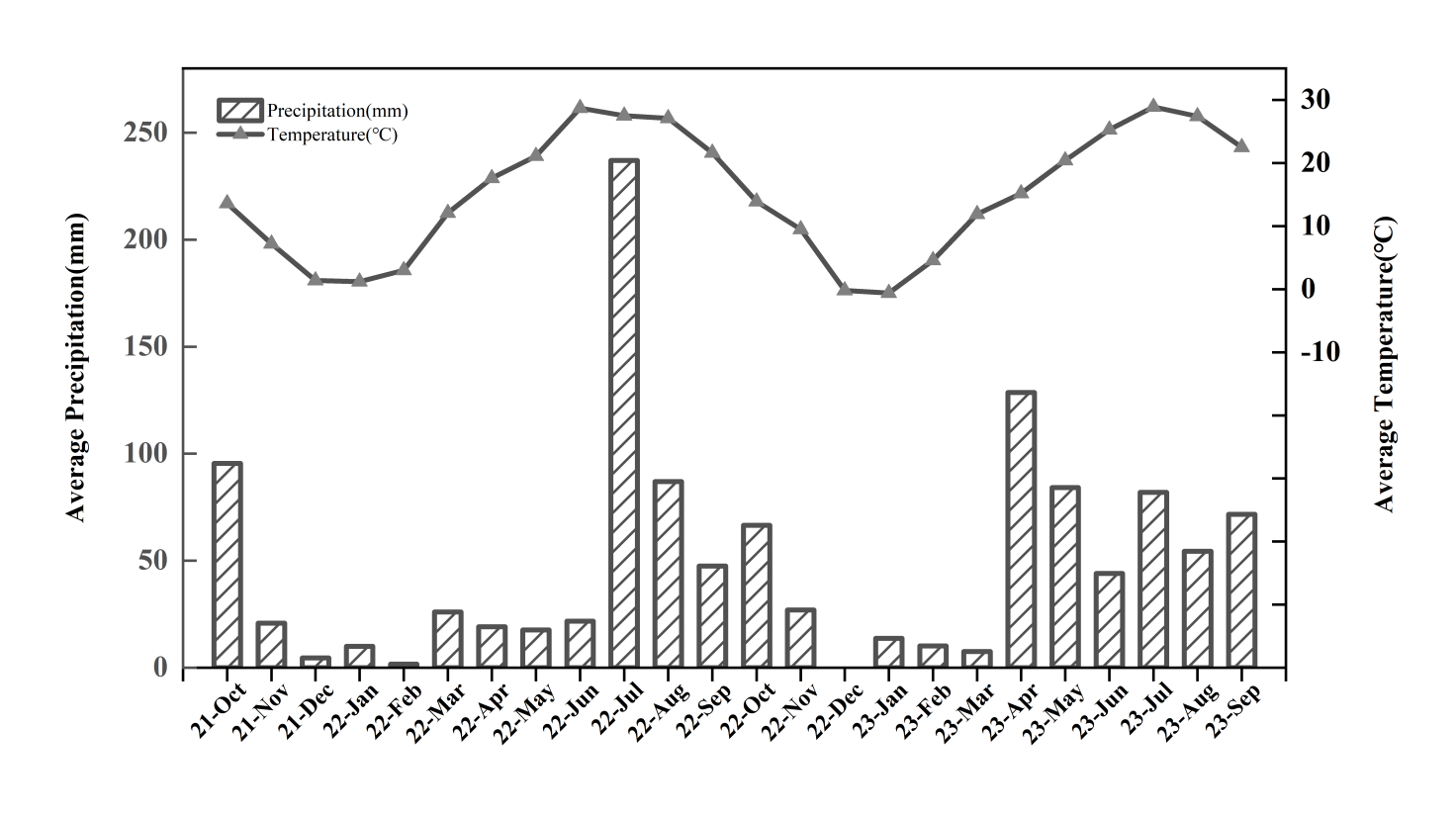
**Figure S1 -** Meteorological data for the years 2021-2023.

**Figure S2 -** OTU-based dilution curves for bacterial and fungal communities. Note: A represent the bacterial dilution curve in the winter wheat season; B represent the fungal dilution curve in the winter wheat season. C represent the bacterial dilution curve in the summer maize season; D represent the bacterial dilution curve in the summer maize season.


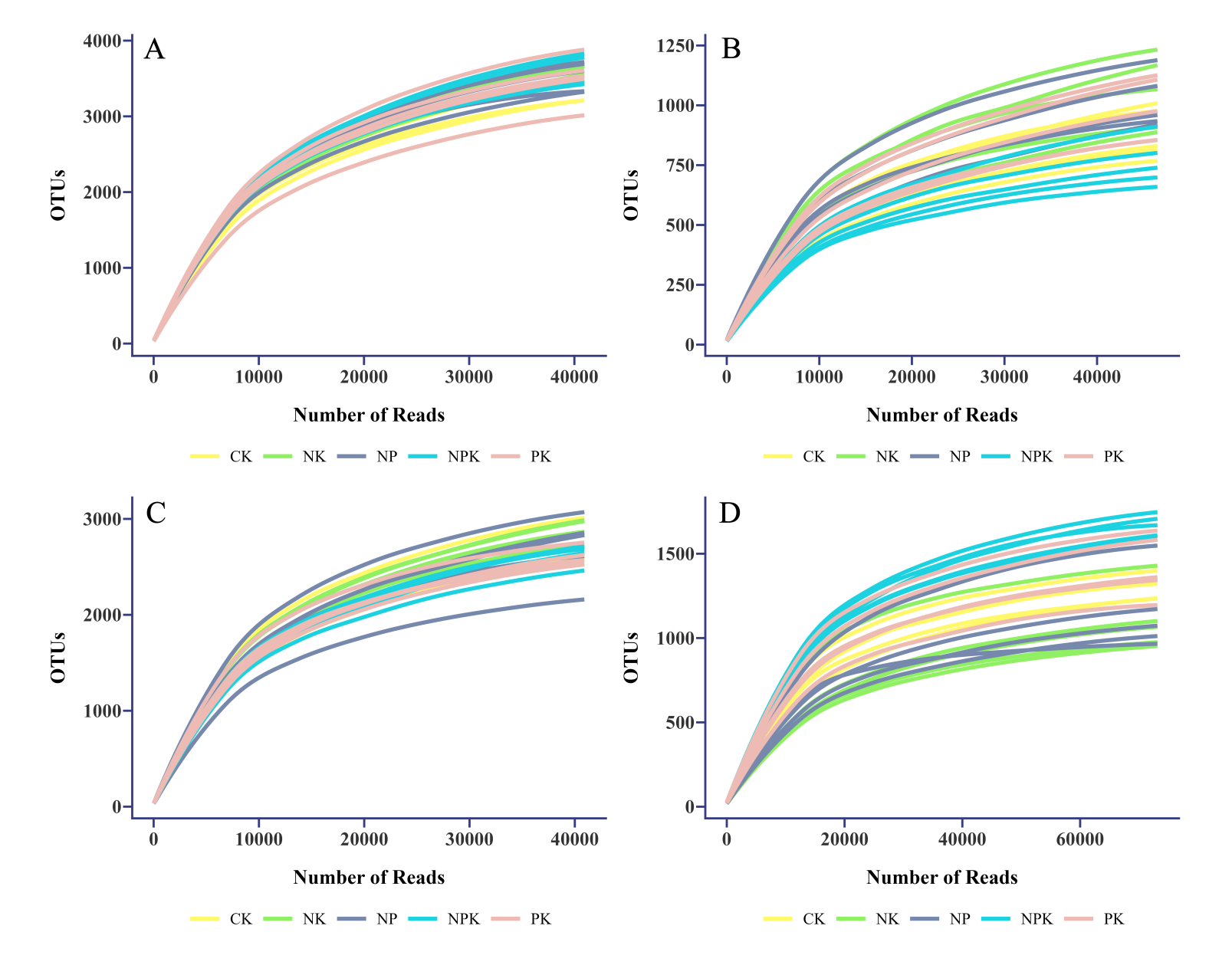

**Figure S3 -** PCoA analysis of soil microorganisms under different nutrient deficiency treatments at phylum level. Note: A represent the winter wheat season bacterial PCoA analysis; B represents the winter wheat fungal PCoA analysis; C represents summer maize bacterial PCoA analysis; D represents the summer maize season fungal PCoA anal.

**Figure S4 -** Effects of different nutrient deficiency treatments on the taxonomic composition of soil bacterial and fungal at the genus level during the winter wheat and summer maize seasons. Note: A represents the top 10 dominant bacterial genera in the winter wheat season; B represents the top 10 dominant fungal genera in the winter wheat season. C represents the top 10 dominant bacterial genera in the summer maize season; D represents the top 10 dominant fungal genera in the summer maize season.


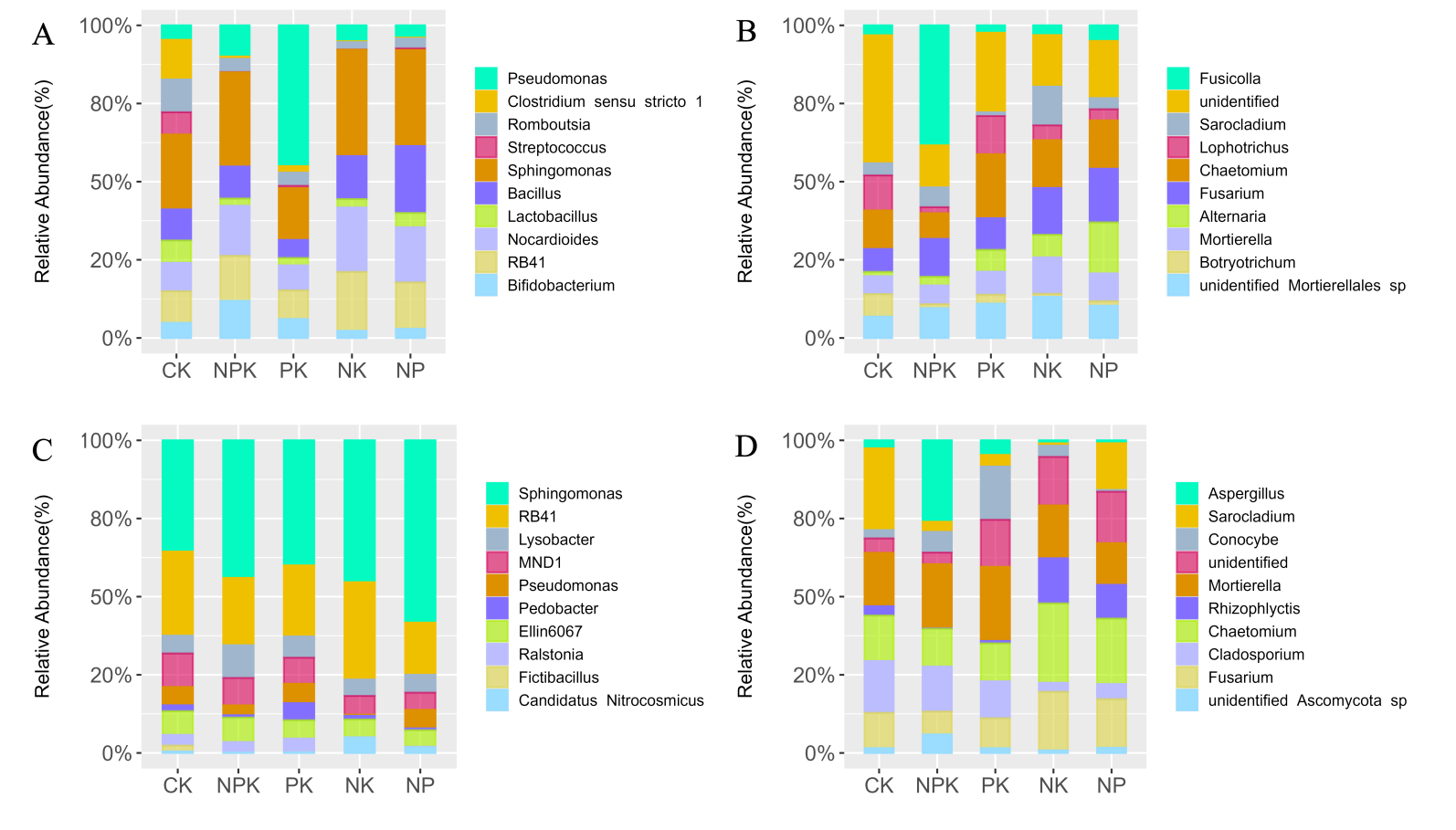


**Figure S5 -** Principal component analysis of functional diversity of different communities. A indicates winter wheat season bacteria; B indicates winter wheat fungi; C indicates summer corn bacteria; D indicates summer corn season fungi analyzed.

**1.2 Supplementary Table**

**Table S1 -** ANOSIM test for soil bacteria and fungi between different nutrient deficiency treatment groups. R^2^ denotes the degree of explanation of the differences of the samples by different subgroups, i.e. the ratio of the variance of different subgroups to the total variance, the larger the R^2^ is, the higher the degree of explanation of group variance is; Pr denotes the P-value, which is less than 0.05, indicating that the test is highly reliable.

| Treatment | Wheat season -bacteria | | Wheat season-fungi | | Maize season -bacteria | | Maize season-fungi | |
| --- | --- | --- | --- | --- | --- | --- | --- | --- |
|  | R2 | Pr（＞F） | R2 | Pr（＞F） | R2 | Pr（＞F） | R2 | Pr（＞F） |
| NPK-PK | 0.11545 | 0.472 | 0.59012 | 0.011 | 0.21836 | 0.007 | 0.19055 | 0.01 |
| NPK-NK | 0.19928 | 0.001 | 0.40962 | 0.001 | 0.44066 | 0.005 | 0.37902 | 0.001 |
| NPK-NP | 0.29282 | 0.001 | 0.56483 | 0.007 | 0.31203 | 0.005 | 0.34069 | 0.012 |
| NPK-CK | 0.22032 | 0.014 | 0.61777 | 0.012 | 0.22534 | 0.009 | 0.17016) | 0.015 |
